# DDX41 and its unique contribution to myeloid leukemogenesis

**DOI:** 10.18632/oncotarget.28603

**Published:** 2024-07-02

**Authors:** Hirotaka Matsui

**Keywords:** acute myeloid leukemia, DDX41, myelodysplastic neoplasms, myeloid neoplasms with germline predisposition, R-loop

Until the early 2000s, myeloid neoplasms attributable to genetic backgrounds were considered exceedingly rare, with notable exceptions limited to those arising as components of systemic syndromes such as Fanconi anemia and Li-Fraumeni syndrome. Historically, no hematopoietic-specific tumor syndromes had been identified until 1999, when *RUNX1* was implicated as the causative gene for familial platelet disorder with a predisposition to acute myeloid leukemia (AML). Subsequently, in 2004, *CEBPA* was recognized as another critical gene responsible for inherited AML.

The subsequent advent and widespread application of comprehensive genetic analysis facilitated the identification of germline pathogenic variants in genes such as *ANKRD26*, *ETV6*, and *GATA2* among patients with myeloid neoplasms that developed against a background of inherited thrombocytopenia or systemic disorders.

It is now established that genetic predisposition is present in approximately 10% of myeloid neoplasms [1], underscoring the fact that myeloid neoplasms with a genetic background are by no means exceptional. According to the 5th edition of the World Health Organization Classification of Haematolymphoid Tumors, myeloid neoplasms with germline predisposition are categorized into 18 distinct syndromes based on disease phenotype and causative genes. However, the number of causative genes is substantially higher, as exemplified by Fanconi anemia, which is caused by numerous genes involved in DNA damage repair.

Among these, myeloid neoplasms caused by *DDX41* variants are particularly noteworthy due to their distinct disease phenotype and pathogenesis [2]. Myelodysplastic neoplasms (MDS) and AML associated with germline pathogenic variants of *DDX41* exhibit a median age of onset of approximately 70 years. This is significantly older than the onset age of myeloid neoplasms with other genetic backgrounds and is comparable to the age of onset in sporadic cases without any apparent genetic predisposition. Moreover, the absence of a distinct phenotype as a hereditary syndrome and the broad spectrum of myeloid neoplasms, ranging from MDS to *de novo* AML, often with hypoplastic features, complicates the inference of a genetic background based solely on clinical presentation. Notably, myeloid neoplasms harboring *DDX41* variants may relapse long after allogeneic hematopoietic stem cell transplantation (HSCT), necessitating vigilance despite the variants not being inherently associated with poor prognosis. Furthermore, instances of secondary leukemia originating from donor cells have been reported in patients who underwent HSCT when the donor harbored the same germline pathogenic variant as the recipient.

The DDX41 protein is an RNA helicase, characterized by a nuclear localization signal at its N-terminus, a DEAD-box domain in the core of the protein, and a helicase domain at its C-terminus. RNA helicases are integral to numerous processes that necessitate the structural conversion of RNA, including RNA splicing, nuclear export of RNA, translation, and ribosomal RNA biogenesis. Accordingly, humans possess a total of 72 distinct RNA helicases, including 41 DEAD-box type, 16 DEAH-box type, and 11 UPF1-like type helicases.

Among these RNA helicases, DDX41 is presumed to function predominantly within the nucleus, although its precise molecular function remains incompletely elucidated. One of the most meticulous studies on DDX41 was published by Chlon et al. in 2021 [3]. They generated mice in which tamoxifen could be used to knock out *Ddx41* and induce a variant corresponding to the human p.R525H, a typical somatic mutation that emerges before overt disease manifestation in cases with a germline variant. The results demonstrated that Ddx41 is an essential factor in mouse hematopoiesis, significantly contributing to ribosomal RNA synthesis via enhanced snoRNA processing.

Our group consistently observed that the *ex vivo* induction of the p.R525H mutation in hematopoietic progenitor cells derived from *Ddx41* heterozygous mice resulted in a rapid decline in proliferative capacity [4]. Furthermore, induction of p.R525H at the organismal level in these mice led to swift hematopoietic failure, thereby underscoring the critical role of DDX41 in hematopoiesis. Notably, our mouse model did not develop myeloid neoplasms, likely due to the pronounced hematopoietic failure phenotype. This limitation highlights the necessity for further refinement of the experimental model.

Another emerging topic in the study of myeloid neoplasms with *DDX41* variants is the accumulation of R-loops. R-loops are known to form during physiological processes, including mitochondrial DNA replication and immunoglobulin class switching. However, they can also accumulate aberrantly in malignancies, including MDS with mutations in genes encoding RNA splicing factors such as *SRSF2* and *SF3B1*, triggering DNA replication stress and genomic instability. In this context, different research groups, including our own, have posited a relationship between *DDX41* variants and R-loop accumulation [4–7]. Nevertheless, it remains unresolved whether DDX41 directly functions as an enzyme that resolves R-loops, or if its inhibited expression or function leads to dysregulation of transcription, RNA splicing, or RNA modification, and therefore, R-loop accumulation.

In summary, myeloid neoplasms associated with *DDX41* variants likely exhibit a unique pathogenesis that diverges from the conventional understanding of myeloid neoplasms. Consequently, further fundamental research is required to establish effective treatment strategies for this specific disease.
